# Performance of next-generation sequencing on small tumor specimens and/or low tumor content samples using a commercially available platform

**DOI:** 10.1371/journal.pone.0196556

**Published:** 2018-04-27

**Authors:** Scott Morris, Janakiraman Subramanian, Esma Gel, George Runger, Eric Thompson, David Mallery, Glen Weiss

**Affiliations:** 1 Paradigm Diagnostics, Phoenix, Arizona, United States of America; 2 Saint Luke’s Health System, Kansas City, Missouri, United States of America; 3 College of Computing, Informatics, and Decision Systems Engineering, Arizona State University, Tempe, Arizona, United States of America; 4 Department of Biomedical Informatics, Arizona State University, Phoenix, Arizona, United States of America; 5 University of Arizona College of Medicine-Phoenix, Phoenix, Arizona, United States of America; CNR, ITALY

## Abstract

**Background:**

Next generation sequencing tests (NGS) are usually performed on relatively small core biopsy or fine needle aspiration (FNA) samples. Data is limited on what amount of tumor by volume or minimum number of FNA passes are needed to yield sufficient material for running NGS. We sought to identify the amount of tumor for running the PCDx NGS platform.

**Methods:**

2,723 consecutive tumor tissues of all cancer types were queried and reviewed for inclusion. Information on tumor volume, success of performing NGS, and results of NGS were compiled. Assessment of sequence analysis, mutation calling and sensitivity, quality control, drug associations, and data aggregation and analysis were performed.

**Results:**

6.4% of samples were rejected from all testing due to insufficient tumor quantity. The number of genes with insufficient sensitivity make definitive mutation calls increased as the percentage of tumor decreased, reaching statistical significance below 5% tumor content. The number of drug associations also decreased with a lower percentage of tumor, but this difference only became significant between 1–3%. The number of drug associations did decrease with smaller tissue size as expected. Neither specimen size or percentage of tumor affected the ability to pass mRNA quality control. A tumor area of 10 mm^2^ provides a good margin of error for specimens to yield adequate drug association results.

**Conclusions:**

Specimen suitability remains a major obstacle to clinical NGS testing. We determined that PCR-based library creation methods allow the use of smaller specimens, and those with a lower percentage of tumor cells to be run on the PCDx NGS platform.

## Introduction

Next generation sequencing (NGS) is increasingly utilized for patients with advanced cancer in an effort to help guide treatment, especially for tumor types that have potential targeted therapy options. Some of the known barriers to successful NGS are tumor tissue adequacy and integrity, and where applicable, availability after routine pathological workup. In some cases, there may also be changes of the tumor’s genomic, transcriptomic, or proteomic profile over time [1]. For patients with unresectable or metastatic disease, NGS is usually performed on relatively small core biopsy or fine needle aspiration samples. Most of the reported data on success rates of sequencing is from lung cancer studies. In a large study from 14 academic centers, only 66% (733/1102) of lung adenocarcinomas could be tested for a panel of 10 genes [2]. In a community practice setting, 11.1% (53/479) of the tissue samples tested for epidermal growth factor receptor (EGFR) and anaplastic lymphoma kinase (ALK) were found to be insufficient [3]. Twenty-three of these 53 patients (43.4%), underwent a second biopsy, and only 70% of the patients that underwent a second biopsy (16/23) were able to get results for both *EGFR* and *ALK*. As reported by another group, NGS testing for 23 genes in lung cancer had a quantity not sufficient (QNS) rate of 33.8% (110/325) [4].

There are known risks associated with tumor tissue retrieval. In addition, costs associated with new tumor tissue acquisition can be prohibitively high, particularly if there is inadequate tissue available for testing. For example, a lung biopsy can run in the order of $14,000 to upwards of over $37,000 if there are complications [3].

In addition, acquisition of new tumor tissue in the case of failed NGS leads to delays in receiving an NGS report and starting the appropriate treatment. Such delays can have negative consequences for a patient [5].

The Paradigm Cancer Diagnostics (PCDx) test is a clinical grade NGS test run in a Clinical Laboratory Improvement Amendments (CLIA)-certified and College of American Pathologists (CAP)-accredited laboratory. The platform measures genomic, transcriptomic, and proteomic aberrations linked with 86 unique therapies based on published patient research information for tumors all cancer types. In addition, the resultant report includes additional clinical trial information related to the biomarkers identified. The mean depth of coverage for DNA copy-number variation is 56,085×, DNA mutation is 13,656×, and RNA is 21,562× [6,7].

We sought to quantify what amount of tumor by volume or minimum number of FNA passes are needed from patients with solid tumors to yield sufficient material for running the PCDx platform.

## Materials and methods

### Study design

We retrospectively collected information on tumor volume, success of performing NGS, and results of NGS for tumor tissue samples that underwent PCDx testing between the years 2014 and 2017. No consent was given, as the data were analyzed anonymously. This work was considered exempt from the requirement for approval since the study was solely reliant on previously obtained and fully anonymized data for which authors did not have access to identifying information. This data set consisted of pass/fail criteria and quality control measures only, and did not contain any identifying information or testing results.

#### Specimen collection

**Fig 1:**
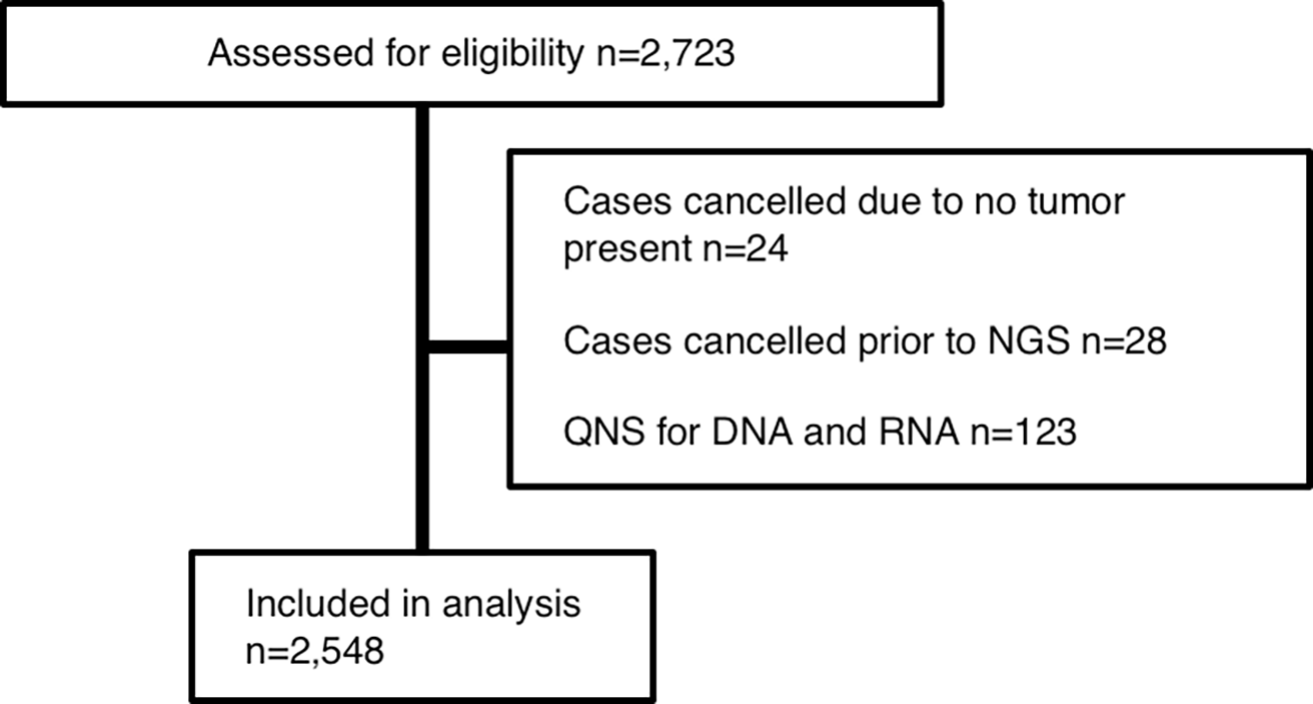
CONSORT diagram. NGS-next generation sequencing, QNS-quantity not sufficient.

A total of 2,723 consecutive patients receiving PCDx testing as part of their clinical care between 2014 and 2017 were included in this analysis (Fig 1).

Twenty-four specimens had their analyses cancelled because there was no tissue remaining in the block received. All diagnoses were accepted, with the most common being non-small cell lung cancer (n = 624), breast cancer (n = 507), colon cancer (n = 222), pancreatic cancer (n = 110) and cancer of unknown primary (n = 129). Formalin-fixed paraffin-embedded (FFPE) blocks were collected and prepared by local procedures within various pathology departments. Shipping containers including a styrofoam container and an ice pack were provided, but specimens were often shipped in different packaging. Most specimens were shipped within the United States by overnight delivery.

#### Specimen testing

Samples were formalin-fixed, paraffin embedded at hospitals by their local protocols. Specimens were shipped by mail, typically using an overnight service. Upon receipt, the tissue within the block was measured along the longest dimension, then a second measurement was taken perpendicular to the first, effectively measuring the smallest rectangle the tissue would fit into. An estimate was made as to the percent of area within the measured rectangle. For example, if the tissue was perfectly rectangular, 100% of the rectangle would be filled with tissue, and if the tissue were a perfect circle, this value would be 0.25*π* = 79%. We use this measurement primarily throughout this study because it is not easy to determine the depth of a specimen prior to selecting a NGS test. Four μm sections were cut for an haematoxylin and eosin (H&E) and immunohistochemical (IHC) staining, and 10 μm tissue sections (“curls”) were cut for DNA and RNA extraction. We attempted to obtain up to 500 mm^2^ of tissue for each nucleic acid type (e.g., if the tissue was 50 mm^2^, we attempted to obtain 10 curls). Frequently this amount was not available, so we used whatever tissue was available. A board-certified pathologist determined the percentage of tumor cell nuclei present on the H&E, and this value was used in sensitivity calculations. IHCs were used for additional tumor characterization, but this aspect is outside the scope of this publication. DNA and RNA were purified and quantified, and libraries were built with PCR and RT-PCR respectively. All reactions were replicated two or four times to maximize accuracy and specificity.

#### Library preparation

DNA was purified with the QIAamp FFPE DNA kit, and RNA was purified with the Roche Highpure kit. cDNA was created from RNA using the QuantiTect reverse transcription kit (Qiagen). Libraries for DNA and RNA were created separately using the Quantifast Multiplex RGQ kit (Qiagen). Reactions were treated with ExoSAP-IT reagent to remove unincorporated primers. A second PCR was conducted to add barcodes to identify patients. Libraries were quantified with the KAPA quantification kit.

#### Sequence analysis

Libraries were sequenced on either the Ion Torrent PGM using a 318 chip or the Illumina NextSeq, using 2x150 chemistry. A custom analysis pipeline was used, and critical components are described in this manuscript. Data was stored in the laboratory information management system (LIMS), and queried for this study. Key features of these algorithms are described below.

#### Mutation sensitivity

All mutation libraries are created in duplicate. Mutations were called when there were at least *c* mutant reads present *and* at least a proportion *r* are mutant in a given library. This condition of calling a mutation implies that the number of mutant reads has to be greater than the maximum of *c* and ⌈*rn*_*i*_⌉, where ⌈.⌉ denotes the ceiling function and *n*_*i*_ is the coverage of a given base in library replicate *i*, where in our study *i* ∈ {1,2} (since all mutation libraries are created in duplicate as stated above). This observation allows us to calculate the probability that a mutation is correctly called in library *i* using the fact that the number of mutant reads can be modeled as a binomial random variable (number of “successes” in *n*_*i*_ trials) where the “success” probability (denoted as *m* below) is equal to the average or expected mutation frequency, usually taken as half the tumor purity. Then, the probability of the event that a mutation is correctly defined in library *i*, which we denote as *T*_*i*_ is equal to the probability of having a number of “successes” that is at least as high as max{*c*,⌈*rn*_*i*_⌉}, which can be calculated as

$$Sensitivity=P(T_{i})=\sum_{k=max\{c,\lceil rn_{i}\rceil \}}^{n_{i}}(\begin{array}{c} n_{i}\\ k \end{array})m^{k}{\left(1-m\right)}^{n_{i}-k}$$

A more powerful sensitivity calculation was used when all reads over a given base were observed to be WT. We define event *W* to be condition that there is at least one mutant observation for the base in question. We define *M* to be the event that there is a mutation. We assume the mutation is present at frequency *r* if we are in state *M*. If we decide we are in *M*^*C*^ when we observe *W*^*C*^, the false negative rate, is *P*(*W*^*C*^|*M*).

$$P(W^{C}|M)=binom(k=0,n,r)=\left(\begin{array}{c} n\\ 0 \end{array}\right)r^{0}{\left(1-r\right)}^{n}={\left(1-r\right)}^{n}$$

We now realize that the sensitivity at this base is simply 1 minus the false negative rate:

$$Sensitivity=1-P(W^{C}|M)=1-{\left(1-r\right)}^{n}$$

If we limit this to only situations where no mutant alleles were observed, we do not need to make any assumptions about error rates so long as we assume that the risk of a mutant allele being incorrectly read as WT, i.e., “wild-type,” is nearly zero.

This calculation provides higher sensitivity than the standard binomial power calculation when the assumptions are met (i.e., all reads of a given base are WT). This is possible because additional information is available in this method (i.e., all reads WT). If the assumptions are not met, the standard binomial power calculation is used on a base-by-base level.

All mutation assays are run in duplicate. Obvious calculations are used to combine replicate sensitivity to overall sensitivity at each base. Once the sensitivity at each base is known, we take a weighted average of the base-level sensitivities. We used COSMIC data from full transcriptomes or genomes to estimate the prior probability of a mutation occurring at each location, and used this value for the weighted average. This provides a single sensitivity value for each gene. If the sensitivity was less than 99% (false negative rate greater than 1%), we reported the gene as having low coverage.

#### Quality control

Copy number variance is defined as the average coefficient of variance between repeated reads of each gene. This variance estimate is used for the statistical test calling significant events. Variance higher than 30% resulted in a rejection of all copy number results.

mRNA variance is defined as the variance between repeated reads of the same gene was determined and compared to variance within the mRNA reference range by a chi-squared test. If the test found a significant difference, all mRNA results were rejected.

Sequencing error was determined by examining the frequency non-reproducible variants identified in only one library replicate. We next estimate the probability that this case contains a false positive.

#### Drug associations

We examined the literature and created a list of rules for recommending treatments. These rules are generally framed as if-then statements, and often involved multiple criteria from IHC, mRNA, copy number and mutation. Findings from each patient were compared to the list of rules, and each criterion met was considered to be a drug association. There were frequently multiple associations found for the same drug. S1 Table displays drug association rules for 20 randomly selected cases.

#### Data aggregation and analysis

We compiled a total of 2,699 consecutive samples with tumor that received NGS as part of their PCDx testing (S2 Table), and compared results with bar graphs for easy visualization. 95% confidence intervals were calculated using Copper-Pearson intervals or standard error of the mean, as appropriate. Analysis of variance and the chi-squared test were used to determine whether significant differences exist in a data set. When a significant difference was found, Tukey’s HSD [8] or chi-squared tests [9] was used to determine which data ranges were different from the highest range (i.e., 25–100% for tumor nuclei and >30mm^2^ for tissue size). An alpha value of 0.05 was used in all statistical tests.

## Results

**Fig 2:**
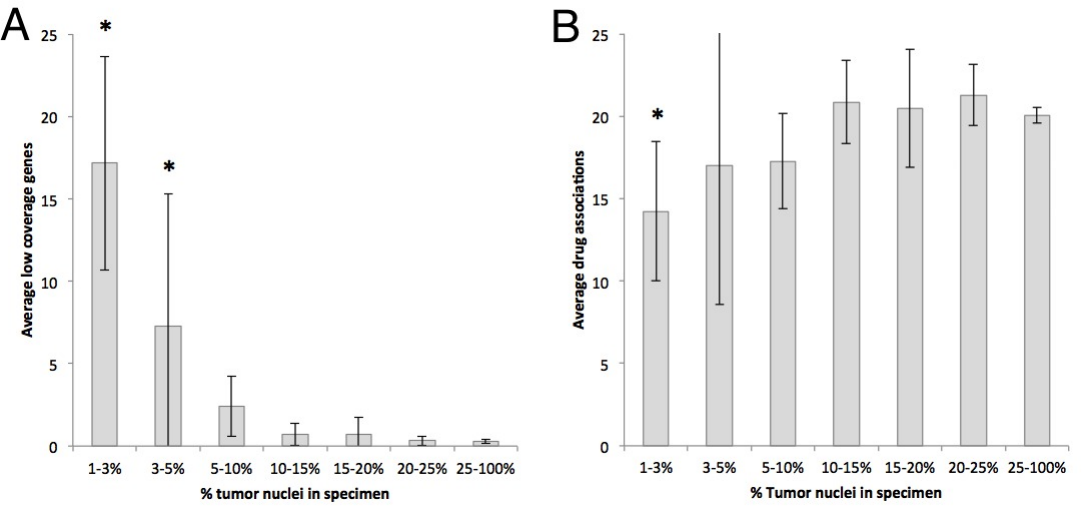
Effects of percentage of tumor nuclei on results. As expected, there were more instances of low coverage (<99% sensitivity) when a lower percentage of tumor nuclei were present (panel A). We observed fewer drug associations on specimens containing 1–3% tumor (panel B).

Overall, only 6.4% (175/2,723) were rejected from all testing due to quantity not sufficient (QNS), including 24 specimens where testing was cancelled before NGS was conducted due to no specimen present in the FFPE block. We examined the effects of the percentage of tumor on results obtained. As expected, the number of low coverage genes increased as the percentage of tumor decreased, with statistical significance below 5% tumor content (Fig 2). The number of drug associations also decreased with a lower percentage of tumor, but this difference only became significant between 1–3% (Fig 2). Even at a low percentage of tumor, many drug associations were found.

**Fig 3:**
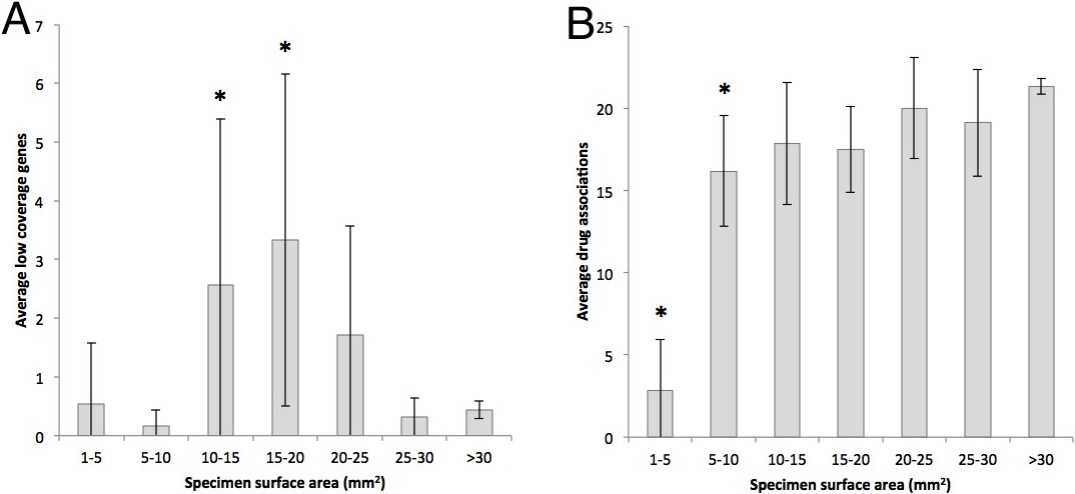
Effects of specimen size on results. Specimens with 10–20 mm^2^ surface area had significantly more low coverage (<99% sensitivity) genes (panel A). There were fewer drug associations identified on the smallest specimens (1–10 mm^2^, panel B).

We next examined the effects of specimen surface area on testing results (Fig 3). Interestingly, the smallest specimens tended to obtain good coverage, but specimens 10–20 mm^2^ had significantly more low coverage than larger specimens. The number of drug associations did decrease with smaller tissue size as expected, with tissues smaller than 20 mm^2^ having a significant decrease when compared to larger tissue sizes, and tissues ≤5 mm^2^ having a dramatic decrease of 21 to 3 drug associations (Fig 3).

**Fig 4:**
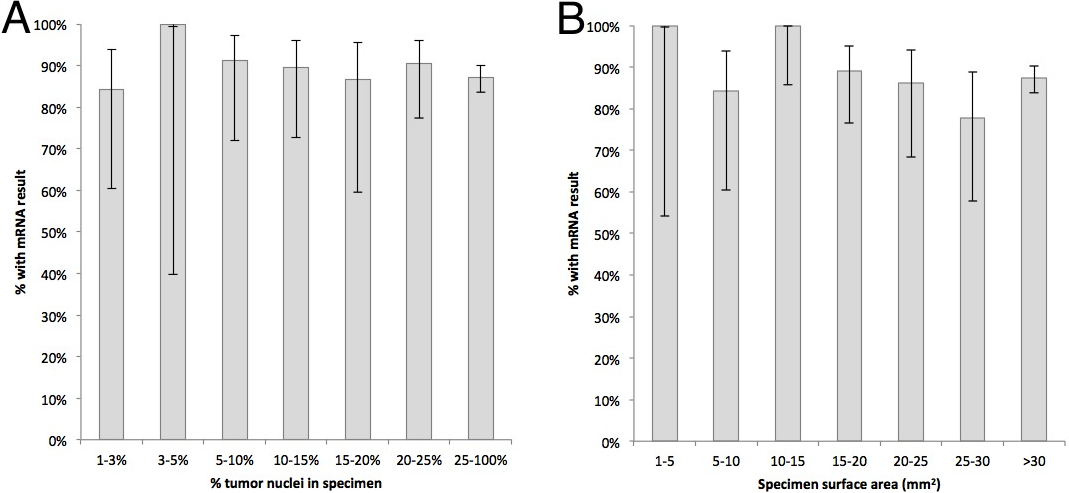
Effects of tumor size and percentage of tumor nuclei on suitability of mRNA for analysis. Note that this measures the ability to detect and normalize mRNA, but does not account for decreases in sensitivity as lower percentage of tumor is present.

Neither specimen size or percentage of tumor affected the ability to pass mRNA quality control and generate a result (Fig 4).

**Fig 5:**
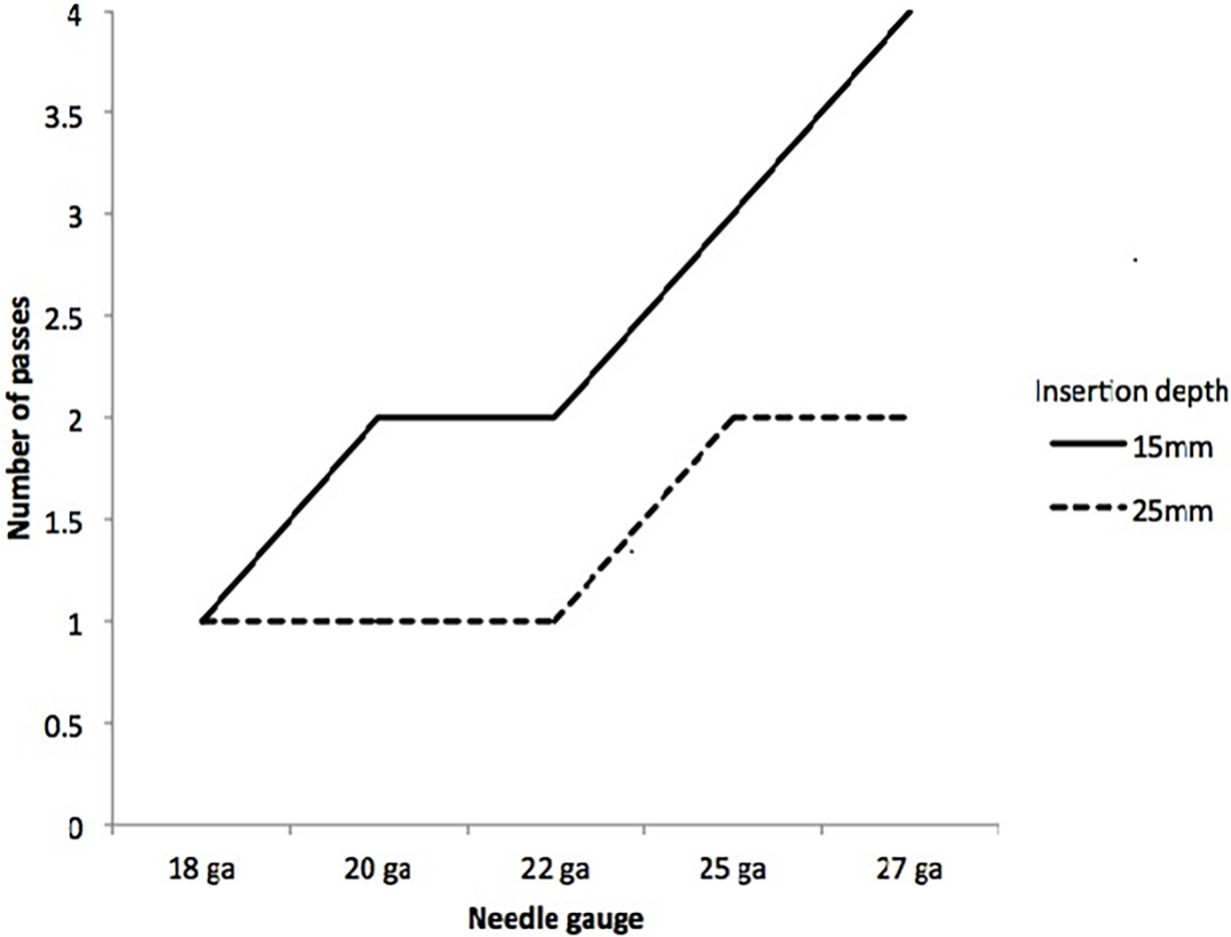
Number of FNA passes vs. needle gauge. This assumes 10 mm^2^ of tissue is required.

We examined Figs 3 and 4 and determined the minimal surface area that could be accepted without statistically significant changes in low coverage genes or in drug association count. Based on these criteria, specimens should be at least 10 mm^2^. Fig 5 explores the number of passes required to obtain 10 mm^2^, based on calculations of surface areas obtained from different needle sizes.

## Discussion

We confirmed that high quality NGS results could be obtained from both small specimens and those with a low percentage of tumor cells in a clinical setting. We had two measures for assessing tumor aberrations: the count of low coverage genes and the number of drug associations found. We found that specimens as small as 10 mm^2^ with 5% tumor could be run without significant decreases in either measure.

The FoundationOne^®^ test has been reported to have a QNS rate of 18% [10]. Here we report that only 6.4% were rejected from all testing due to QNS. The ultimate goal of molecular profiling is to identify potential drug candidates and contraindications, so we believe drug associations is a better measure. By this measure, the PCDx platform can still yield drug association results in specimens with tumor cell percentage as low as 3%. There may be a slight increase in low coverage genes. However, while genes are classified as low coverage when the sensitivity is <99%, they are still identifiable and reported as an indeterminant result. Overall, the number of drug associations does not appear to be affected with 3% tumor nuclei (Fig 2). One could argue that the tumor area could be lowered to 5 mm^2^ based on Fig 3, since adequate drug association results were still obtained in the 5–10 mm^2^ range. However, setting the limit at 10 mm^2^ gives a better margin for error in the common situation where there are discrepancies in measurements between institutions procuring and shipping tissue specimens.

It should be noted that tissue area is an imperfect measure. Tissues are three-dimensional, and the volume, not surface area, is what is ultimately important. We were unable to find a simple and non-destructive method to determine the depth of a tissue that could be performed by standard pathology departments. We concluded that surface area is still the best method because it can be readily measured with a ruler in a few seconds, and decided it was best to focus this study on an imperfect but practical measure that would be readily used on future cases.

The only unexpected result was the relationship between specimen surface area and low coverage genes. One would expect smaller specimens to have more low coverage genes, but we found the most low coverage in samples of intermediate size range. We verified with lab personnel that no special handling was conducted on these samples. Post hoc analysis of laboratory documents revealed that small specimens tended to have more curls cut from them. In other words, the three dimensional shape was not the same as other specimens and the smaller samples tended to extend much deeper into the block. The decrease in actionable results below 5 mm^2^ was likely due to excessive paraffin being provided to nucleic acid extraction as more curls were cut from smaller tissues.

Limitations of this study include its retrospective nature, and to a lesser extent, smaller sample sizes within some experimental groups.

Today, much of the current NGS testing performed both at the research level and by commercial sources is primarily restricted to measuring genomic aberrations. This work shows the benefit of more global testing of DNA, RNA, and protein to identify potential therapeutic targets. PCDx was recently prospectively evaluated in a single center study, and 43% of patients treated with NGS guided therapy attained a progression-free survival (PFS) ratio ≥1.3 versus 5% treated with non-genomically guided therapy (p<0.0001) [11]. PFS ratio ≥1.3 has been used as a criterion to demonstrate that the selected therapy has favorably altered the expected natural course of advanced disease [12]. In advanced cancer, successive lines of therapy would normally be expected to have decreased PFS, unless a therapy is altering the disease course whereby the PFS is increased (ideally in a clinically meaningful way with little to no toxicity) [13]. Interestingly, 8/19 (42%) of targets mainly identified by mRNA expression would have been missed because these targets are absent from assays that do not measure gene expression.

It is well known that many tumors are heterogenous, not only morphologically, but also molecularly. Tumor can consist of multiple distinct genomic subclones and these subclones may be present at low frequency [14]. The threshold for detection of variants using Sanger sequencing is about 10% prevalence. The use of more sophisticated PCR strategies that involve enrichment for low frequency populations can yield thresholds of detection as low as 0.1% prevalence [14]. Here, we show that the PCDx platform may identify low frequency subclones that might be missed by other NGS platforms. The ability to detect mutations down to 3% tumor nuclei in specimens is indifferent from the ability to detect mutations in a subclone that makes up 3% of the specimen.

Specimen suitability remains a major obstacle to clinical NGS testing. We determined that PCR-based library creation methods allow the use of smaller specimens, and those with lower percentage of tumor cells.

## Supporting information

S1 Table Drug association rules for 20 randomly selected cases. (XLSX)

S2 Table Complete dataset. (XLSX)
