# Correction: Performance of next-generation sequencing on small tumor specimens and/or low tumor content samples using a commercially available platform

**DOI:** 10.1371/journal.pone.0200224

**Published:** 2018-06-28

**Authors:** Scott M. Morris, Janakiraman Subramanian, Esma S. Gel, George C. Runger, Eric J. Thompson, David W. Mallery, Glen J. Weiss

Middle initials are missing from the names of the first, third, fourth, fifth, sixth, and seventh authors. The complete, correct names are: Scott M. Morris, Esma S. Gel, George C. Runger, Eric J. Thompson, David W. Mallery, Glen J. Weiss. The correct citation is: Morris SM, Subramanian J, Gel ES, Runger GC, Thompson EJ, Mallery DW, et al. (2018) Performance of next-generation sequencing on small tumor specimens and/or low tumor content samples using a commercially available platform. PLoS ONE 13(4): e0196556. https://doi.org/10.1371/journal.pone.0196556
